# Assessment of UHF Frequency Range for Failure Classification in Power Transformers

**DOI:** 10.3390/s24155056

**Published:** 2024-08-05

**Authors:** Karl Schiewaldt, Bruno Albuquerque de Castro, Jorge Alfredo Ardila-Rey, Marcelo Nicoletti Franchin, André Luiz Andreoli, Stefan Tenbohlen

**Affiliations:** 1 School of Engineering, Bauru, Department of Electrical Engineering, São Paulo State University (UNESP), Bauru 17033-360, SP, Brazil; karl.schiewaldt@unesp.br (K.S.); marcelo.franchin@unesp.br (M.N.F.); andre.andreoli@unesp.br (A.L.A.); 2 Department of Electrical Engineering, Universidad Técnica Federico Santa María, Av. Vicuña Mackenna 3939, Santiago de Chile 8940000, Chile; jorge.ardila@usm.cl; 3 Institute of Power Transmission and High Voltage Technology (IEH), University of Stuttgart, 70569 Stuttgart, Germany; stefan.tenbohlen@ieh.uni-stuttgart.de

**Keywords:** pattern recognition, partial discharges, electric arc, UHF, power transformers

## Abstract

Ultrahigh-frequency (UHF) sensing is one of the most promising techniques for assessing the quality of power transformer insulation systems due to its capability to identify failures like partial discharges (PDs) by detecting the emitted UHF signals. However, there are still uncertainties regarding the frequency range that should be evaluated in measurements. For example, most publications have stated that UHF emissions range up to 3 GHz. However, a Cigré brochure revealed that the optimal spectrum is between 100 MHz and 1 GHz, and more recently, a study indicated that the optimal frequency range is between 400 MHz and 900 MHz. Since different faults require different maintenance actions, both science and industry have been developing systems that allow for failure-type identification. Hence, it is important to note that bandwidth reduction may impair classification systems, especially those that are frequency-based. This article combines three operational conditions of a power transformer (healthy state, electric arc failure, and partial discharges on bushing) with three different self-organized maps to carry out failure classification: the chromatic technique (CT), principal component analysis (PCA), and the shape analysis clustering technique (SACT). For each case, the frequency content of UHF signals was selected at three frequency bands: the full spectrum, Cigré brochure range, and between 400 MHz and 900 MHz. Therefore, the contributions of this work are to assess how spectrum band limitation may alter failure classification and to evaluate the effectiveness of signal processing methodologies based on the frequency content of UHF signals. Additionally, an advantage of this work is that it does not rely on training as is the case for some machine learning-based methods. The results indicate that the reduced frequency range was not a limiting factor for classifying the state of the operation condition of the power transformer. Therefore, there is the possibility of using lower frequency ranges, such as from 400 MHz to 900 MHz, contributing to the development of less costly data acquisition systems. Additionally, PCA was found to be the most promising technique despite the reduction in frequency band information.

## 1. Introduction

Science and industry have been striving to develop ultrahigh-frequency (UHF) technologies for detecting early-stage failures caused by dielectric degradation in power transformers in order to enhance maintenance planning through the identification and classification of faults by sensing the electromagnetic waves emitted by partial discharges, electric arcs, corona, etc. Although UHF is a traditional methodology applied to high-voltage maintenance assets, there are uncertainties about which frequency range should be evaluated in a measurement [1]. Most studies have shown that UHF emissions vary within 3 GHz. However, a Cigré brochure revealed that the optimum spectrum is between 100 MHz and 1 GHz [2]. More recently, a study indicated that the range from 400 MHz to 900 MHz is enough for successful measurements [1].

In this sense, the restriction of the frequency band, as suggested by [1], is a key factor in reducing the cost of data acquisition systems. On the other hand, the lack of frequencies may limit the information that supplies frequency-based pattern recognition tools. Among several data classification methods, self-organized maps such as the chromatic technique (CT) [3,4] and principal component analysis (PCA) maps [5,6,7] stand out for not depending on a prior training process. However, data limitation may impair the capability to perform pattern recognition since the data input depends on the frequency content of the signals.

There are a lot of scientific and technological challenges in the power transformer monitoring field, one of them being that different failures require different maintenance actions. Therefore, although the UHF technique is widely applied to failure detection, it is important to develop pattern recognition systems that improve the capabilities of the maintenance plans to avoid machine outages. The goal of this work is to assess how the frequency band limitation may interfere with failure classification in power transformers and also provide a comparative study of three self-organized maps applied to classify three types of transformer condition failure. The pattern recognition tools are the chromatic technique (CT) [3,4], principal component analysis (PCA) [7,8], and the shape analysis clustering technique (SACT), which combined the kurtosis and skewness [9] as a 2D-mapping system of the spectrum of transformer’s failures. As opposed to [10,11], in which the PCA technique is combined with artificial intelligence like the support vector machine (SVM) or multi-variable k-nearest neighbors, in this work, the frequency content of the UHF signals feed and enhance the PCA technique in order to perform spectrum-based self-organized maps.

The contributions of this work are summarized as follows:

- Assess how the spectrum band limitation may alter the failure classification for frequency-based pattern recognition tools. The chosen ranges are full signal, from 100 MHz to 1 GHz (suggested by Cigré brochure [2]), and from 400 MHz to 900 MHz (suggested by Tenbohlen et al. 2023 [1]).
- Provide a comparison of three self-organized methods to perform failure classification.
- Classify three operational conditions: transformer with partial discharges in insulators, electric arcs inside the transformer, and healthy operation.

This article is divided into six sections. A brief discussion about transformer failures is discussed in Section 2, followed by an explanation of the pattern recognition tools used in this work presented in Section 3. Section 4 shows the method and experimental setup. Results and discussions are presented in Section 5. Section 6 ends the article with the conclusions.

## 2. Failures and UHF Emissions in Power Transformers

Power transformers are often exposed to several types of failures due to operational or environmental conditions, which may lead to dielectric degradation. According to Soni and Mehta (2021) and Tenbohlen et al. (2017) [12,13], between 38% and 48% of transformer failures occur in the windings, followed by 30% and 15% in tap changers and bushings, respectively. Due to the thermal energy produced by overloads, various cellulose-based materials such as press-boarders, kraft papers, or the enamel on wires may decompose and recombine to form new chemical products with lower dielectric strength [14]. In the case of the windings, according to Murugan and Ramasamy (2019) [15] the conductor tilting, conductor bending, and axial instability due to careless transportation or vibration may generate spots without insulation that culminate in short circuits or electric arcs inside the power transformer. Chemical recombination, moisture, and particle penetration also increase dielectric degradation, leading to partial discharge (PD) activity, which is defined by the IEC 60270 [16] as a discharge that does not completely bridge two conducting parts, being an ionization process. Another transformer part that suffers PD activity is the insulator bushing, since, in many cases, its performance is influenced by polluting particles, and dirt commonly present in the environment, industry, coastal areas, etc. which decreases the dielectric strength over time [17,18]. Partial discharges and electric arcs frequently disappear and then reappear, producing heating, ultraviolet radiation, acoustic emission, and electromagnetic waves [19]. According to Azirani et al. (2021) [19], the accelerated motion of electric charges in the PD or arcs leads to the generation of both an electric and a magnetic field, fulfilling the fundamental requirement for the radiation of electromagnetic waves as stated by Poynting’s theorem [20].

Based on this behavior, one of the most promising transformer failure detection techniques is the use of UHF antennas for detecting the signals emitted by the flaws. However, as discussed in the previous section, there are uncertainties about which UHF frequency range should be evaluated [1]. In addition, several technological issues must be taken into account. For example, different flaws require different maintenance actions. In this scenario, several studies have tried to develop failure localization or classification systems. For example, Do et al. (2020) [21] applied a convolutional neural network to classify six failures with a UHF range of 1.8 GHz. The failures were protruding electrode, floating, particle, surface PD, turn-to-turn discharges, and void PD. Jangjoo, Allahbakhshi, and Mirzaei (2023) performed a PD localization system based on the time of arrival of the UHF antenna with a range of up to 3 GHz [22]. Internal and creep discharges were studied by Jiang et al. (2021) [11], with a bandwidth of sensors between 300 MHz and 2 GHz. In Cheng et al. (2021) [23], an antenna with a range from 200 MHz to 800 MHz was combined with a high-frequency current transform (HFCT) to perform the identification of four types of failures: protrusion, floating metal, insulation air void, and insulation surface. Skewness, kurtosis, and energy parameters were the input to an artificial neural network to classify three types of defects in transformers by using measurements up to 2 GHz in [24]. Although these techniques have proven to be effective, it is important to conduct a more in-depth analysis of the frequency bands of UHF signals and to develop classification techniques that do not require supervised training. Therefore, the next sections will introduce the signal processing analysis used to classify three operational conditions of the transformer.

## 3. Signal Processing Analysis

As mentioned, the spectrum content reduction may impact the amount of information on the UHF signals that feed frequency-based pattern recognition systems. The objective of this paper is to assess the effectiveness of three types of pattern recognition techniques by applying three different frequency bands.

In order to obtain the frequency content of the UHF signals emitted by the transformer’s flaws, given a discrete UHF sequence x[n] with *N* samples, the discrete Fourier transform (DFT) X[k] is defined as follows:

(1) $$X\left[k\right]=\sum_{n=0}^{N-1}x\left[n\right]e^{-j\frac{2\pi kn}{N}}$$

(2) $$f\left[k\right]=\frac{2\pi k}{N},k=0,1,2,\ldots ,N-1$$

where f[k] is the frequency.

In this work, the DFT was calculated from all UHF signals considering all the transformer’s operational conditions. After that, for each case, the chromatic technique (CT), the shape analysis clustering technique (SACT), and the principal component analysis (PCA) were applied as data separation systems.

The CT and SACT are characterized as effective feature extraction tools which allow effective pattern recognition from complex signals without the need for a supervised training process. The objective is to apply each parameter as a Cartesian system [3,4].

For the CT, the most important frequency-based parameters are the average band (AB) and the equivalent bandwidth (EB), defined as follows:

(3) $$AB=\frac{\sum_{k=0}^{N-1}f\left[k\right]\left|X[k]\right|}{\sum_{k=0}^{N-1}\left|X[k]\right|},$$

In this work, AB represents the simple weighted average of the frequency content and EB the effective bandwidth (RMS bandwidth) of a given signal:

(4) $$EB=\sqrt{\frac{\sum_{k=0}^{N-1}f{\left[k\right]}^{2}{\left|X[k]\right|}^{2}}{N.E}}.$$

where *E* is the energy of a signal, defined as:

(5) $$E=\frac{1}{N}\sum_{k=0}^{N-1}{\left|X\left[k\right]\right|}^{2}.$$

The chromatic technique (CT) is applied as a data reduction tool, as the discrete Fourier transform vector is converted into two parameters that serve as coordinates in a 2D Cartesian system (EB × AB). In this study, the CT was also used as a self-organizing map to assess whether the frequency band limitation impacts the effectiveness of the technique.

The shape analysis clustering technique (SACT) is based on the skewness and kurtosis statistics. The objective is to perform data separation maps by using these statistics to assess the behavior of the shape of the spectrum of all cases. In this scenario, kurtosis (K) and skewness (S) [9] are used as a bi-dimensional coordinate system and they are defined as:

(6) $$S=\sum_{n=0}^{N-1}{\left[\frac{|X[k]|-\mu }{\sigma }\right]}^{3}$$

where μ is the average amplitude of the |X[k]| and σ is the standard deviation of |X[k]|.

In this case, skewness can measure the symmetry of the frequency content, indicating if the failure has more left or right spectrum.

Kurtosis (K) is used as a coordinate parameter aiming to verify the degree of peakedness of the frequency content:

(7) $$K=\sum_{n=0}^{N-1}{\left[\frac{|X[k]|-\mu }{\sigma }\right]}^{4}$$

The purpose is to use these statistics to perform a classification of the operational condition of the power transformer by identifying the pattern of the shapes of each failure.

Regarding principal component analysis (PCA), according to Simon Haykin (2009) [25]: “PCA transformation is designed in such a way that the data set may be represented by a reduced number of effective features yet retain most of the intrinsic information content of the original data; in other words, the data set undergoes a dimensionality reduction”. In this scenario, the selected data are the matrix constituted by all absolute values of the discrete Fourier transforms (|X[k]|) related to all operational conditions of the transformer. Considering each failure pattern, it can be concluded that the data are dispersed in each DFT, indicating high variance across |X[k]|. The goal after PCA is to concentrate the data variance in the principal components, for which, in this work, we considered the first two principal components (PC1 and PC2). To calculate these ’parameters, given a matrix, in this case, constituted by all absolute values of the DFTs related to all operational conditions of the transformer $X_{M}$ and its covariance matrix $COV_{X_{M}}$, principal components 1 and 2 (PC1 and PC2) were extracted by using the singular value decomposition, carried out by [5,6,7]:

(8) $$COV_{X_{M}}=USU^{T}$$

where *U* consists of an orthonormal matrix containing the eigenvectors of $COV_{X_{M}}$ and *S* is a diagonal matrix. According to the technique, the first two columns of *U* are called the dominant eigenvectors or the principal components 1 (PC1) and 2 (PC2). In this article, PC1 and PC2 were applied as a two-dimensional coordinate system in order to verify the capability of the PCA technique to distinguish the three operational conditions of a power transformer.

## 4. Method

In this section, the experimental setup is presented, followed by the description of the signal analysis applied in this work.

### 4.1. Experimental Setup

**Figure 1:**
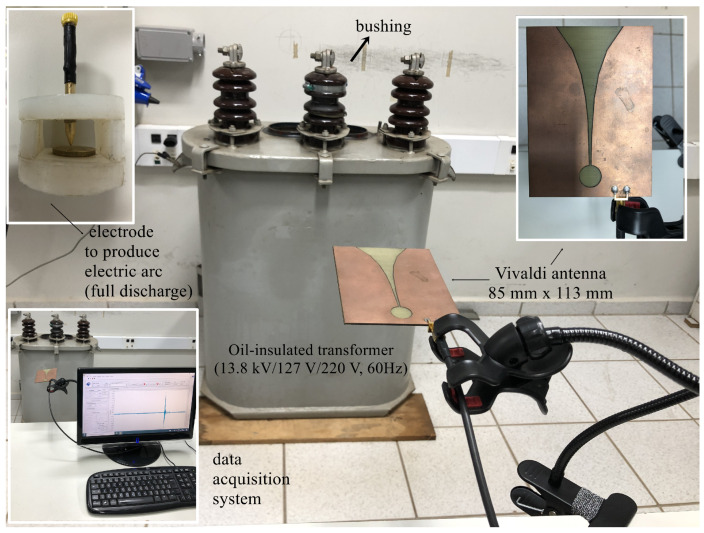
Experimental setup.

In order to extract UHF signals from power transformer failures, several tests were carried out in a 30 kVA oil-insulated distribution transformer (13.8 kV/127 V/220 V, 60 Hz). The aim was to assess three operational conditions: the machine without failure, with partial discharge activity on its insulator, and with electric arcs (known as full discharges) inside the transformer’s core. To generate the first fault, graphite powder was deposited on the bushing in order to emulate surface contamination, which, in practical scenarios, can be caused by dirt, bird feces, etc. A high-voltage source supplied the bushing with 13 kV. To generate the full discharges in the transformer’s core, a copper-based electrode with a gap of 2 mm, supplied by 3 kV, was immersed in the transformer’s tank. In practical applications, this type of flaw may occur due to spots of insulation losses caused by chemical deterioration, winding conductor bending, axial instability, winding movement, etc. A Vivaldi antenna, made from a circuit board with dimensions of 85 mm × 113 mm, was positioned 1 m away from the electrical machine and its signals were acquired using a dedicated digital to analog converter for the data acquisition system (M4i.2233-x8 from SPECTRUM Instrumentation^®^, Grosshansdorf, Germany). The acquisition rate was set at 2.5 GHz. Figure 1 shows the experimental setup.

**Figure 2:**
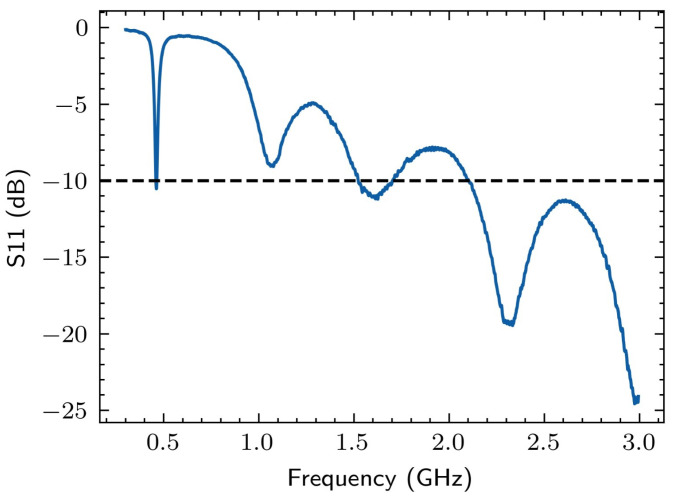
Reflection parameter of the Vivaldi antenna.

The reflection parameter of the Vivaldi antenna (S11) measured with a commercial vector network analyzer (VNA)—similar to [26]—is illustrated in Figure 2.

It can be verified that the antenna can capture spectral components across the entire UHF range, with greater efficiency in the frequency range between 400 and 2400 MHz. The electromagnetic emission of the PD pulses is approximately in the range of 300 MHz to 1.0 GHz, making the antenna suitable for measuring this phenomenon. In this scenario, 100 tests of each type of failure (bushing, full discharge, and no failure) were carried out.

### 4.2. Failure Classification Analysis

As the aim of this work is to evaluate the efficiency of three frequency bands in classifying the operational condition of the transformer, the signals were conditioned in three ways: first, without filtering; second, with a digital 4th-order Butterworth band-pass filter from 100 MHz to 1 GHz (according to the range proposed by Cigré technical brochure 861 [2]); and third, with the same band-pass filter from 400 MHz to 900 MHz (according to the range indicated by [1]).

The Fourier transform was extracted for each signal, forming a matrix of spectra for the three conditions studied: transformer with full discharge, transformer with partial discharge in the insulator, and transformer without fault but with the antenna capturing the background noise. The first and last 50 points of the absolute value of DFT were excluded to mitigate boundary influences, DC level, or low-frequency waves. After that, the spectrum was normalized. With this matrix, the chromatic technique was applied, and each spectrum was summarized in a two-dimensional coordinate system formed by the equivalent bandwidth and average band statistics.

**Figure 3:**
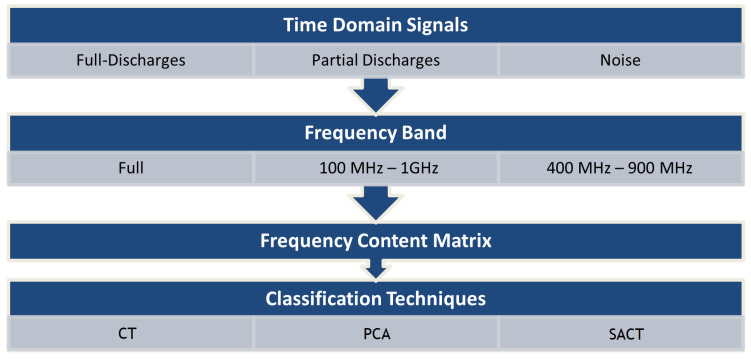
Flowchart of signal processing analysis.

After that, the principal component analysis (PCA) was applied to the entire spectrum matrix, extracting the first two principal components as a coordinate system. Finally, the shape analysis clustering technique (SACT) was carried out by calculating the kurtosis and skewness of each matrix line. Figure 3 shows a flowchart of the signal processing methodology.

The coordinate systems were created in order to form two-dimensional maps to assess the classification capability of each pattern recognition methodology, considering the three frequency ranges studied.

## 5. Results and Discussion

This section is organized into five subsections. First, it presents signals in both the time and frequency domains. Subsequently, it discusses the results of failure classification techniques for each frequency range (full spectrum, Cigré TB 681 (100 MHz–1 GHz) [2], and Tenbohlen et al. (2023) [1] (400 MHz–900 MHz). After that, a brief comparative analysis is given.

### 5.1. Time and Frequency Analysis

**Figure 4:**
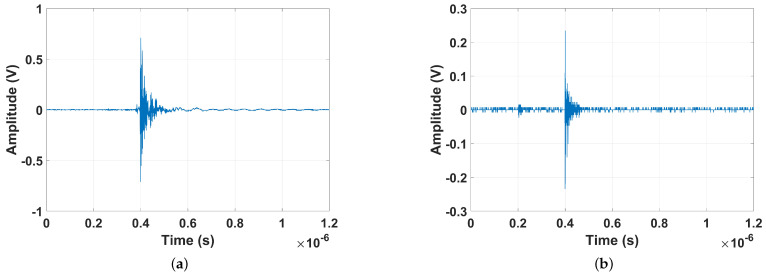
Time-domain signals from (**a**) full discharge (electric arch) and (**b**) partial discharge on insulator.

In Figure 4, the UHF signals in the time domain produced by internal full discharges (electric arches) and partial discharges on the insulator are presented.

**Figure 5:**
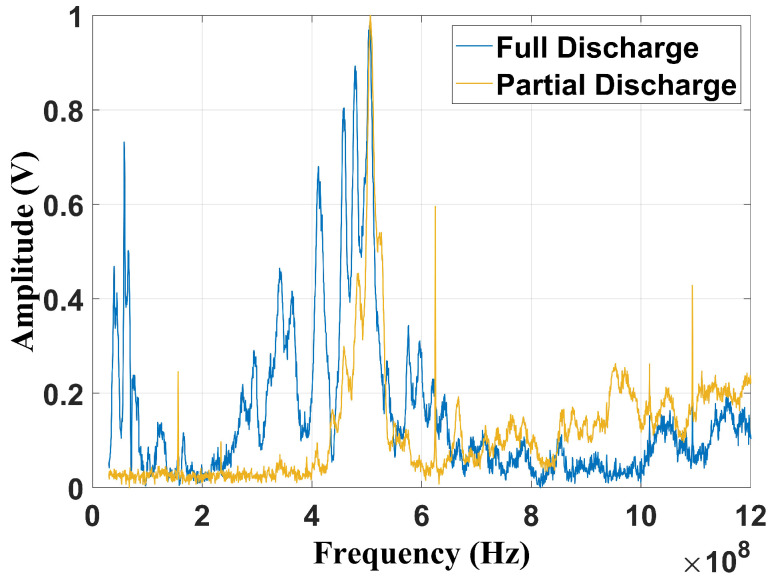
Frequency content of full and partial discharges, considering the raw UHF signal.

By analyzing the signals, it can be seen that both faults have impulsive behavior that lasts around 0.2 µs. The peak value for the partial discharge on the insulator was 0.23 V, and for the full discharge, 0.7 V. Figure 5 presents the normalized average spectrum of each failure type. Both flaws have peaks at 500 MHz. However, for the full discharge, the frequency spectrum has significance between 300 MHz and 600 MHz and also presents amplitudes by 100 MHz (VHF (very high frequency) band). On the other hand, for the partial discharge, the peaks are concentrated between 420 MHz and 600 MHz.

**Figure 6:**
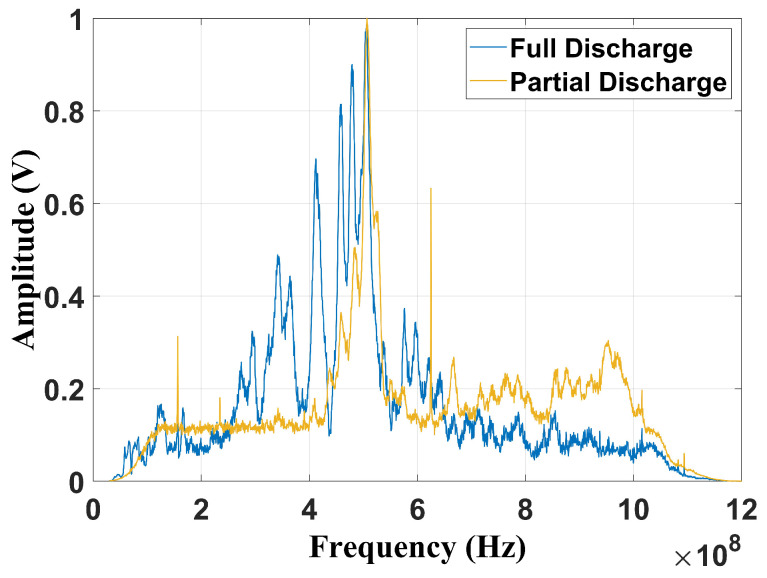
Frequency content filtered from 100 MHz to 1 GHz.

**Figure 7:**
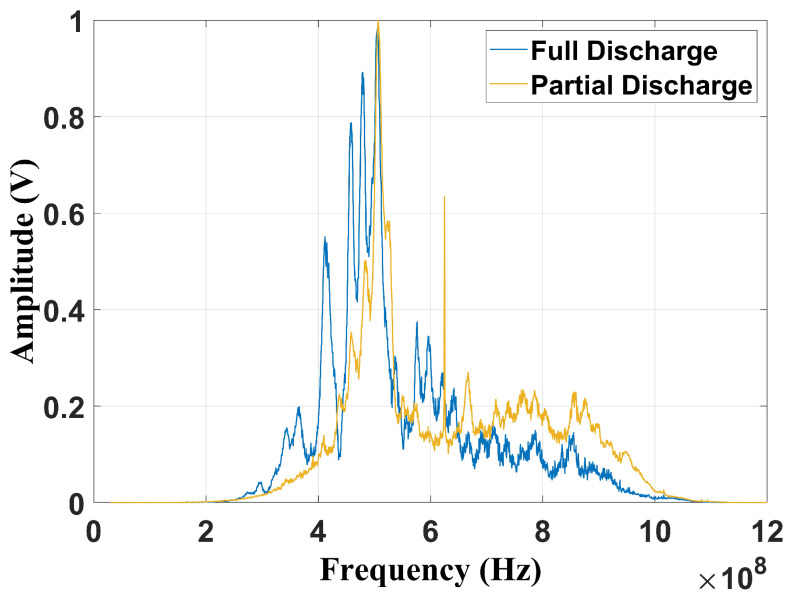
Frequency content filtered from 400 MHz to 900 MHz.

Figure 6 and Figure 7 present, respectively, the frequency content of each failure considering the frequencies suggested by Cigré TB 861 [2] (100 MHz–1 GHz) and by Tenbohlen et al. (2023) [1] (400 MHz–900 MHz). The signatures were obtained using a digital fourth-order Butterworth band-pass filter implemented in MATLAB (2022b).

**Figure 8:**
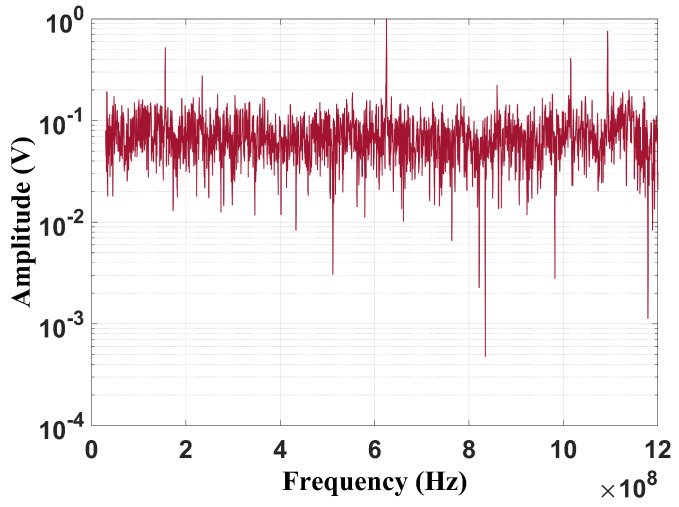
Frequency content of the background noise.

By the analysis of Figure 6, it can be verified that the peak value is also at 500 MHz, with the full discharges being more dispersed than the partial discharge fault. Nevertheless, considering the frequency band suggested by [1], due to the frequency limitation, both spectra present significant similarities (Figure 7). Based on this issue, it is important to evaluate how the reduction in the frequency content may affect fault classification, especially for techniques that depend on frequency information. In this study, three classification methods were evaluated: the chromatic technique (CT), principal component analysis (PCA), and shape analysis clustering technique (SACT). Three operational conditions were considered: the two mentioned flaws and the background UHF noise, emulating the transformer without failure. The goal was also to asses whether noise can cause false diagnoses. The background noise spectrum is shown in Figure 8.

It can be observed that the noise remains constant across the entire frequency range, even in the bands where the discharges are characterized. In addition, it is important to note that measurements performed in the laboratory may contain signals from other sources, such as radio and television transmissions, represented by the peaks observed at 180 MHz, 620 MHz, 1.05 GHz, and 1.1 GHz. Therefore, this noise is present in all measurements, as in real scenarios.

### 5.2. Chromatic Technique

**Figure 9:**
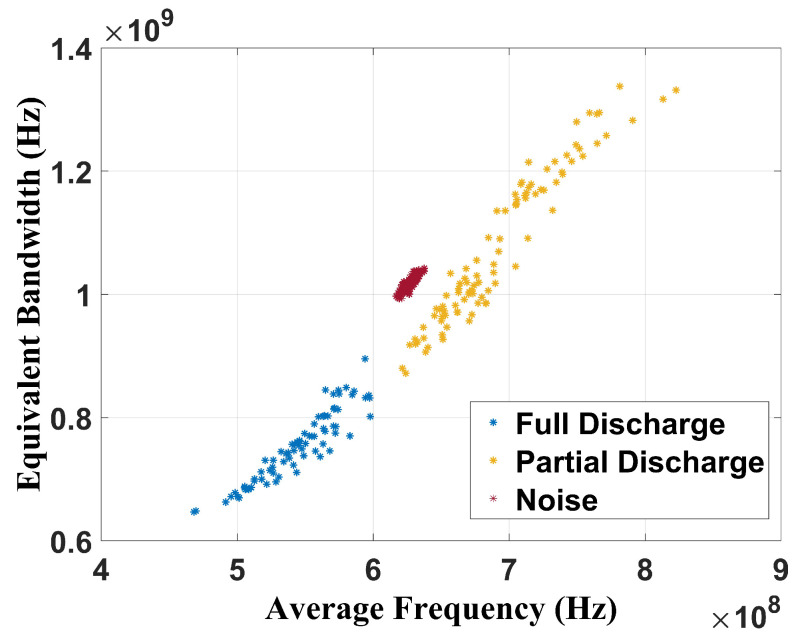
Chromatic technique for raw UHF signals.

Figure 9 shows the chromatic technique (CT) applied to the full-band signal, without filtering.

**Figure 10:**
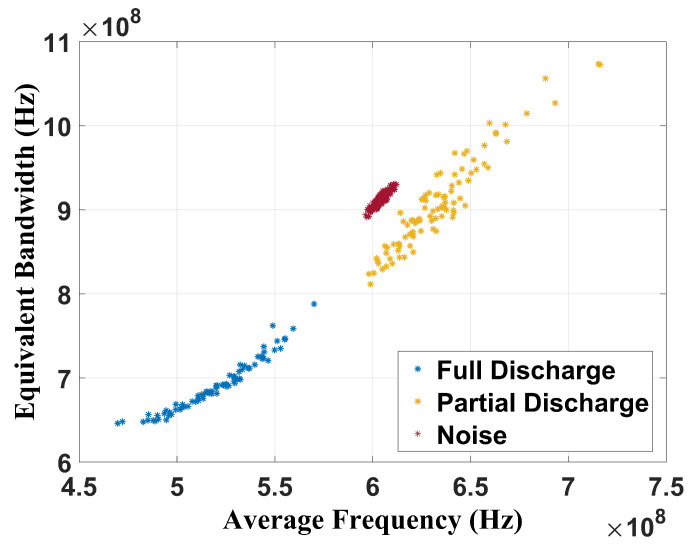
Chromatic technique for frequency band from 100 MHz to 1 GHz.

The analysis of these results shows that the CT is effective in performing failure classification since three well-defined regions related to full discharge, partial discharge, and no failure (only noise) state can be observed. For the full discharge, the average band was between 460 MHz and 600 MHz and the equivalent bandwidth was from 640 MHz to 900 MHz. For the partial discharges, the average band ranged from 630 MHz to 830 MHz and the equivalent bandwidth from 700 MHz to 1.30 GHz. For the background noise, the CT showed average values of 630 MHz for the average band and 1.05 GHz for the equivalent band. Figure 10 shows the chromatic technique (CT) applied to the 100 MHz–1 GHz frequency range (Cigré range).

**Figure 11:**
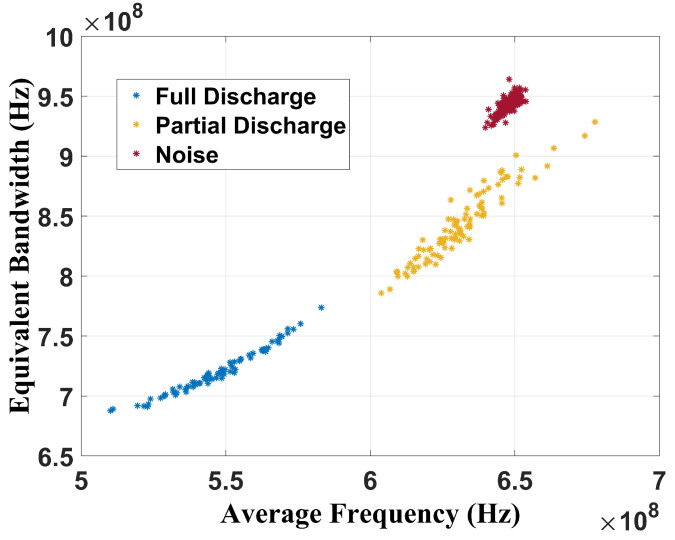
Chromatic technique for frequency band from 400 MHz to 900 MHz.

The chromatic technique was also effective in classifying the faults since there are three well-defined regions. For the full discharge, the average band was between 460 MHz and 580 MHz, and the equivalent bandwidth was between 650 MHz and 800 MHz. For the partial discharge, the average band ranged from 600 MHz to 720 MHz and the equivalent bandwidth from 800 MHz to 1.08 GHz. The CT showed values of 610 MHz for the average band and 910 MHz for the equivalent bandwidth when the transformer was without failure. Figure 11 presents the CT applied to signals between 400 MHz and 900 MHz (Tebohlen et al. (2023) [1] range).

The graphical analysis also shows three well-defined regions separating the operational conditions of the power transformer. For full discharges, the average band values were between 510 MHz and 590 MHz and between 680 MHz and 770 MHz for the equivalent band. For partial discharges, the values were 600 MHz to 660 MHz for the average band and 790 MHz to 930 MHz for the equivalent bandwidth. The no failure condition had average values of 645 MHz for the average band and 950 MHz for the equivalent bandwidth. Hence, it can be concluded that the chromatic technique was effective in performing condition classification, regardless of the frequency range assessed.

### 5.3. PCA Clustering Technique

**Figure 12:**
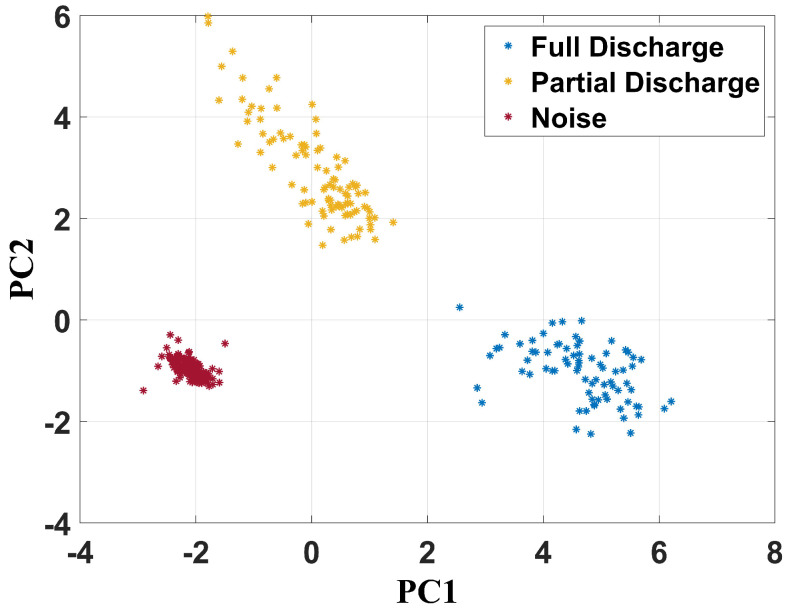
PCA clustering technique for unfiltered UHF signals.

As mentioned, principal component analysis (PCA) was applied to the frequency content matrix formed by the spectrum of all signals extracted from all operational conditions studied in this work. Therefore, two-dimensional maps were created by using principal component 1 (PC1) and principal component 2 (PC2) as a Cartesian system. Figure 12 shows the PCA as a data separation technique for the unfiltered signals.

It can be verified that PCA was effective in achieving failure classification since there are three well-established clusters, characterized by the partial discharge, full discharge, and noise points. Concerning the full discharges, PC1 ranged from 2.5 to 6.5 and PC2 from −2.5 to 0. For the partial discharges, PC1 and PC2 were between −2 and 1.5 and 1.5 and 6, respectively. The cluster related to the health condition measurements had its center at −2 and −1 for PC1 and PC2, respectively.

**Figure 13:**
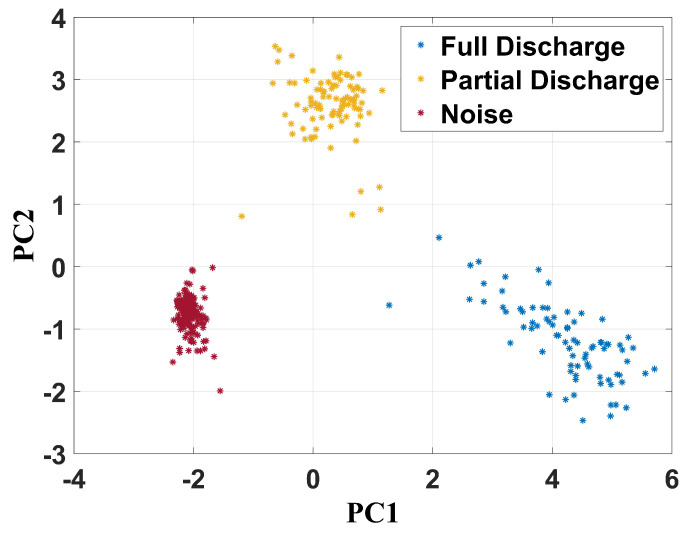
PCA clustering technique for frequency band from 100 MHz to 1 GHz.

For the signals filtered from 100 MHz to 1 GHz (Figure 13), PC1 ranged from 1 to 6 and PC2 from −2.5 to 0.5 for full discharges. Concerning the partial discharge activity, PC1 and PC2 were between −1.2 and 1.1 and 0.8 and 3.5, respectively. The group of measurements of the health operational condition had their center at −2 and −1.1 for PC1 and PC2, respectively.

**Figure 14:**
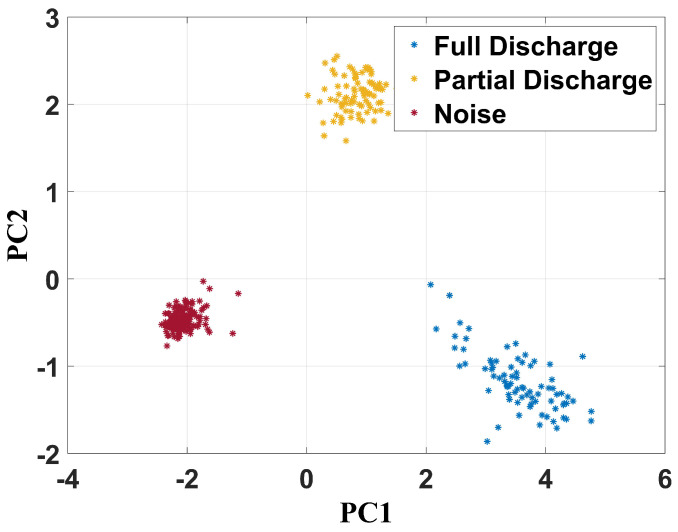
PCA clustering technique for frequency band from 400 MHz to 900 MHz.

Figure 14 shows the same analysis for the frequency band between 400 MHz and 900 MHz.

Although the frequency range was smaller, three well-defined regions can be noted in the figure, demonstrating the capability of the technique to perform data separation. The noise points (without failure) were centered at −2 and −0.5 for PC1 and PC2, respectively. PC1 varied between 0 and 1.5 and PC2 between 1.5 and 2.6 for partial discharge. In relation to the full discharge, PC1 and PC2 varied between 2 and 5 and between 0 and −2, respectively.

### 5.4. Shape Analysis Clustering Technique

**Figure 15:**
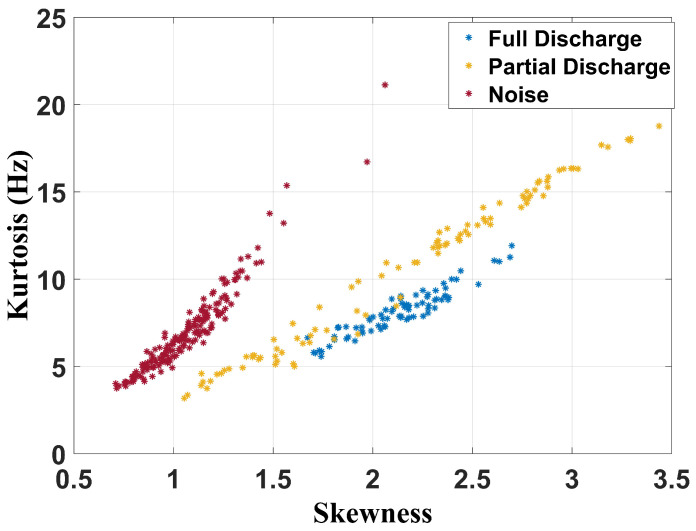
Shape analysis clustering technique for full frequency band.

**Figure 16:**
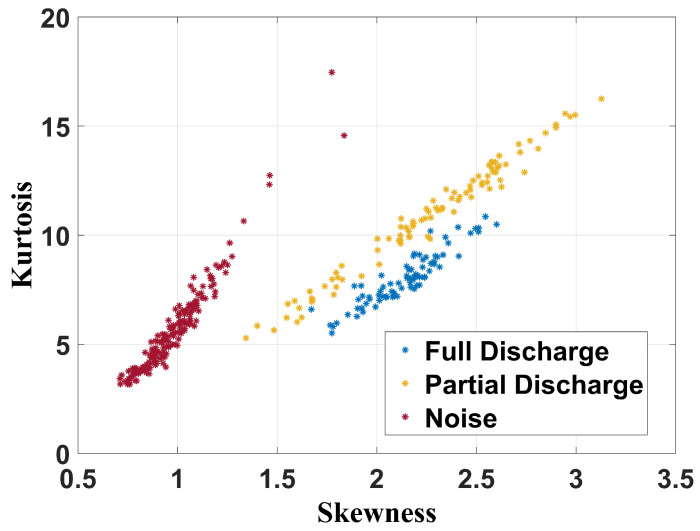
Shape analysis clustering technique for frequency band from 100 MHz to 1 GHz.

**Figure 17:**
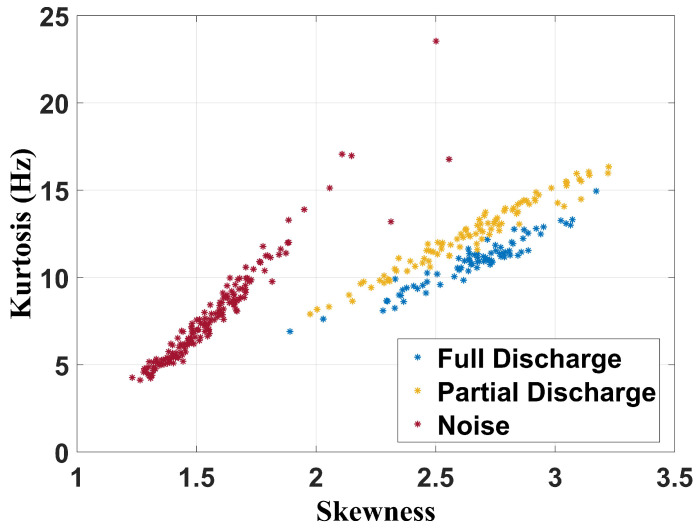
Shape analysis clustering technique for frequency band from 400 MHz to 900 MHz.

Figure 15, Figure 16 and Figure 17 show the separation maps formed by the shape analysis clustering technique regarding, respectively, the full frequency band, 100 MHz to 1 GHz band, and 400 MHz to 900 MHz spectrum.

Based on the analysis of the results, it can be concluded that shape analysis was not effective in characterizing the failures, as there are common features in the regions associated with both partial and full discharges. However, this technique can effectively classify whether a transformer has a fault, as the cluster associated with noise measurements—when the transformer is in a healthy state—is distinctly separated from the clusters related to the faults.

### 5.5. Comparative Analysis

**Figure 18:**
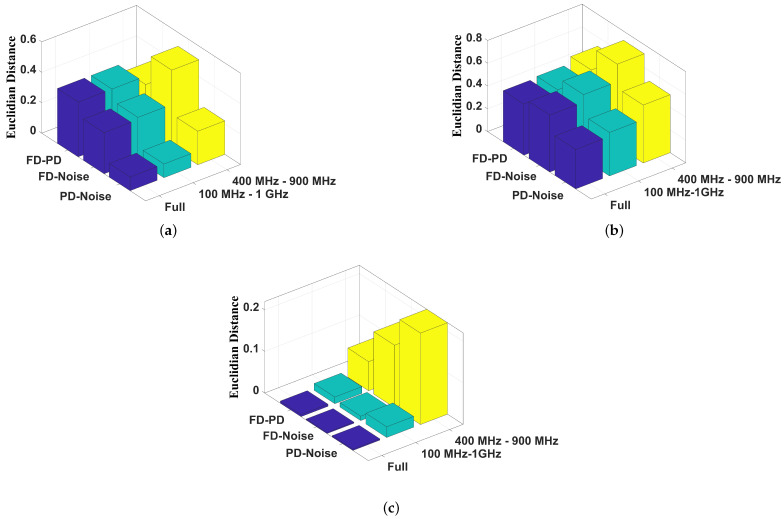
Euclidean distance from each cluster from (**a**) chromatic technique, (**b**) PCA, and (**c**) shape analysis (FD — full discharge cluster, PD — partial discharge cluster and Noise — no failure cluster).

To further compare the classification techniques, the Euclidean distances between the geometric centers of each cluster were extracted (Figure 18). For Euclidean distance calculation, the Cartesian points were normalized since each technique has its own dimension.

For the chromatic technique, Figure 18a shows that the Euclidean distances between the clusters related to the full (FD) and partial discharges (PD) were 0.36, 0.37, and 0.31 units for the full band, the 100 MHz to 1 GHz band, and the 400 MHz to 900 MHz band, respectively, indicating that the limitation of the frequency band did not impair the failure classification. For the same pattern recognition technique, the distances of the clusters formed by partial discharges (PD) and noise were 0.1, 0.09, and 0.22 units for, respectively, the full band, the 100 MHz to 1 GHz band, and the 400 MHz to 900 MHz band. For the full discharge (FD) and noise (no failure condition), the distance was 0.27 units for the full band, 0.29 for the 100 MHz to 1 GHz band, and 0.51 units for the 400 MHz to 900 MHz spectrum.

The PCA technique proved to be the most promising pattern recognition technique since it presented the highest distance values (Figure 18b). Considering the clusters formed by PD and FD, the distances were, respectively, 0.45, 0.47, and 0.52 units for the full band, the 100 MHz to 1 GHz band, and the 400 MHz to 900 MHz band. Regarding the PD and measurements without fault, the values were 0.34, 0.38, and 0.51 units for the full band, the 100 MHz to 1 GHz band, and the 400 MHz to 900 MHz band, respectively. For FD and noise, the distances were 0.50 units for the full band, 0.56 for the 100 MHz to 1 GHz band, and 0.72 units for the 400 MHz to 900 MHz spectrum. In this context, the frequency band limitation improves the effectiveness of the operational condition classification.

The shape clustering technique showed the smallest distance between the clusters. Figure 18c shows that the Euclidean distances between the clusters related to the full and partial discharges were 0.005, 0.02, and 0.07 units for the full band, the 100 MHz to 1 GHz band, and the 400 MHz to 900 MHz band, respectively. For the same pattern recognition technique, the distances of the clusters formed by the PD and noise were 0.003, 0.03, and 0.20 units for, respectively, the full band, the 100 MHz to 1 GHz band, and the 400 MHz to 900 MHz band. For the FD and noise, the distances were 0.002 units for the full band, 0.01 for the 100 MHz to 1 GHz band, and 0.14 units for the 400 MHz to 900 MHz spectrum.

In this scenario, shape analysis presented lower Euclidean distances, and PCA, combined with the frequency band between 400 MHz and 900 MHz, proved to be the best pattern recognition technique studied in this work. In addition, the limitation of the frequency band allows low-cost UHF data acquisition systems.

## 6. Conclusions

The literature review revealed that the frequency spectrum of UHF for transformer failure measurements is, according to the Cigré brochure, from 100 MHz to 1 GHz and, more recently, bands between 400 MHz and 900 MHz. In this scenario, the limitation of the frequency band is a key factor in reducing the cost of implementing data acquisition systems that may demand high acquisition rate frequencies. On the other hand, spectrum band limitation may limit the frequency information that supplies frequency-based pattern recognition systems. Based on this issue, this work assesses three transformer fault classification techniques considering three cases: full band, frequencies from 100 MHz to 1 GHz (Cigré TB 861 [2]) and from 400 MHz to 900 MHz [1]. The results indicated that the reduced frequency range was not a limiting factor for fault classification and identification, and the PCA proved to be the most promising failure classification technique. It is important to note that none of the pattern recognition tools proposed in this work require prior training; instead, these techniques can self-organize. Additionally, this research demonstrates that the frequency range analysis can be restricted without compromising failure classification. This finding is significant for reducing the costs of implementing UHF data acquisition systems. Nevertheless, future work can consider new antenna topologies, signal processing techniques, new transformer faults, and superimposed failure signals.

## Data Availability

Further inquiries can be directed to the corresponding author.

## References

[1] Tenbohlen S., Beura C.P., Sikorski W., Albarracín Sánchez R., Castro B.A., Beltle M., Fehlmann P., Judd M., Werner F., Siegel M. (2023). Frequency range of UHF PD measurements in power transformers. Energies.

[2] Tenbohlen S., Coenen S., Siegel M., Linn T., Markalous S., Mraz P., Beltle M., Naderian A., Schmidt V., Fuhr J. (2022). Improvements to PD measurements for factory and site acceptance tests of power transformers. Cigre Technical Brochure.

[3] Wang X., Li X., Rong M., Xie D., Ding D., Wang Z. (2017). UHF signal processing and pattern recognition of partial discharge in gas-insulated switchgear using chromatic methodology. Sensors.

[4] Ardila-Rey J.A., Schurch R., Poblete N.M., Govindarajan S., Muñoz O., Castro B.A. (2021). Separation of Partial Discharges Sources and Noise Based on the Temporal and Spectral Response of the Signals. IEEE Trans. Instrum. Meas..

[5] Granato D., Santos J.S., Escher G.B., Ferreira B.L., Maggio R.M. (2018). Use of principal component analysis (PCA) and hierarchical cluster analysis (HCA) for multivariate association between bioactive compounds and functional properties in foods: A critical perspective. Trends Food Sci. Technol..

[6] Liu X., Renard C.M., Bureau S., Le Bourvellec C. (2021). Revisiting the contribution of ATR-FTIR spectroscopy to characterize plant cell wall polysaccharides. Carbohydr. Polym..

[7] Castro B.A., Binotto A., Ardila-Rey J.A., Fraga J.R.C.P., Smith C., Andreoli A.L. (2023). New Algorithm Applied to Transformers’ Failures Detection Based on Karhunen-Loève Transform. IEEE Trans. Ind. Inform..

[8] Wang Z., Tian G., Meo M., Ciampa F. (2018). Image processing based quantitative damage evaluation in composites with long pulse thermography. Ndt E Int..

[9] Demir S. (2022). Comparison of normality tests in terms of sample sizes under different skewness and Kurtosis coefficients. Int. J. Assess. Tools Educ..

[10] Iorkyase E.T., Tachtatzis C., Atkinson R. (2023). PCA-enhanced methodology for the identification of partial discharge locations. Energies.

[11] Jiang J., Chen J., Li J., Yang X., Bie Y., Ranjan P., Zhang C., Schwarz H. (2021). Partial discharge detection and diagnosis of transformer bushing based on UHF method. IEEE Sens. J..

[12] Soni R., Mehta B. (2021). Review on asset management of power transformer by diagnosing incipient faults and faults identification using various testing methodologies. Eng. Fail. Anal..

[13] Tenbohlen S., Jagers J., Vahidi F. Results of a standardized survey about the reliability of power transformers. Proceedings of the 20th International Symposium on High Voltage Engineering.

[14] Gamez C. (2018). Dissolved gas analysis, measurements and interpretations. Power Transformer Condition Monitoring and Diagnosis.

[15] Murugan R., Ramasamy R. (2019). Understanding the power transformer component failures for health index-based maintenance planning in electric utilities. Eng. Fail. Anal..

[16] (2000). High-Voltage Test Techniques—Partial Discharge Measurements.

[17] Hashemnia N., Abu-Siada A., Islam S. (2016). Detection of power transformer bushing faults and oil degradation using frequency response analysis. IEEE Trans. Dielectr. Electr. Insul..

[18] Yang Z., Jiang X., Zhang Z., Zhang D., Liu Y. (2016). Study on the influence rules of soluble contaminants on flashover voltage of disc suspension insulators. IEEE Trans. Dielectr. Electr. Insul..

[19] Azirani M.A., Ariannik M., Werle P., Akbari A. (2021). Optimal frequency selection for detection of partial discharges in power transformers using the UHF measurement technique. Measurement.

[20] Pozar D.M. (2021). Microwave Engineering: Theory and Techniques.

[21] Do T.D., Tuyet-Doan V.N., Cho Y.S., Sun J.H., Kim Y.H. (2020). Convolutional-neural-network-based partial discharge diagnosis for power transformer using UHF sensor. IEEE Access.

[22] Jangjoo M.A., Allahbakhshi M., Mirzaei H.R. (2023). UHF sensors positioning on the power transformer tank to enhance the partial discharge localization accuracy. Electr. Power Syst. Res..

[23] Cheng J., Xu Y., Ding D., Liu W. (2020). Investigation of the UHF partial discharge detection characteristics of a novel bushing tap sensor for transformers. IEEE Trans. Power Deliv..

[24] Sinaga H.H., Phung B., Blackburn T.R. (2014). Recognition of single and multiple partial discharge sources in transformers based on ultra-high frequency signals. IET Gener. Transm. Distrib..

[25] Haykin S. (2009). Neural Networks and Learning Machines.

[26] Ardila-Rey J.A., Figueroa D., Torres F.P., Govindarajan S., Castro B.A., Schurch R. (2024). Bioinspired Ultra High Frequency Antenna for Partial Discharge Detection in High-Voltage Equipment. IEEE Trans. Instrum. Meas..

